# Applying Next-Generation Sequencing to Track HIV-1 Drug Resistance Mutations Circulating in Portugal

**DOI:** 10.3390/v16040622

**Published:** 2024-04-17

**Authors:** Victor Pimentel, Marta Pingarilho, Cruz S. Sebastião, Mafalda Miranda, Fátima Gonçalves, Joaquim Cabanas, Inês Costa, Isabel Diogo, Sandra Fernandes, Olga Costa, Rita Corte-Real, M. Rosário O. Martins, Sofia G. Seabra, Ana B. Abecasis, Perpétua Gomes

**Affiliations:** 1 Global Health and Tropical Medicine (GHTM), Associate Laboratory in Translation and Innovation Towards Global Health (LA-REAL), Instituto de Higiene e Medicina Tropical (IHMT), Universidade NOVA de Lisboa (UNL), Rua da Junqueira 100, 1349-008 Lisbon, Portugal; martapingarilho@ihmt.unl.pt (M.P.); cruzsebastiao@ihmt.unl.pt (C.S.S.); mafaldansmiranda@ihmt.unl.pt (M.M.); mrfom@ihmt.unl.pt (M.R.O.M.); sgseabra@ihmt.unl.pt (S.G.S.); ana.abecasis@ihmt.unl.pt (A.B.A.); 2 Centro de Investigação em Saúde de Angola (CISA), Caxito, Angola; 3 Instituto Nacional de Investigação em Saúde (INIS), Luanda, Angola; 4 Laboratório de Biologia Molecular, Serviço de Patologia Clínica, Unidade Local de Saúde Lisboa Ocidental, Hospital Egas Moniz, 1349-019 Lisbon, Portugal; mfmgoncalves@chlo.min-saude.pt (F.G.); jcabanas@chlo.min-saude.pt (J.C.); icosta@chlo.min-saude.pt (I.C.); Ifmadeira@chlo.min-saude.pt (I.D.); smfernandes@chlo.min-saude.pt (S.F.); pcrsilva@chlo.min-saude.pt (P.G.); 5 Biologia Molecular, Serviço de Patologia Clínica, Centro Hospitalar de Lisboa Central, 1150-199 Lisbon, Portugal; ompena@netcabo.pt (O.C.); cortereal.rita@gmail.com (R.C.-R.); 6 Egas Moniz Center for Interdisciplinary Research (CiiEM), Egas Moniz School of Health & Science, Caparica, 2829-511 Almada, Portugal

**Keywords:** HIV-1, drug resistance, INSTIs, NGS, Portugal, Europe

## Abstract

Background: The global scale-up of antiretroviral treatment (ART) offers significant health benefits by suppressing HIV-1 replication and increasing CD4 cell counts. However, incomplete viral suppression poses a potential threat for the emergence of drug resistance mutations (DRMs), limiting ART options, and increasing HIV transmission. Objective: We investigated the patterns of transmitted drug resistance (TDR) and acquired drug resistance (ADR) among HIV-1 patients in Portugal. Methods: Data were obtained from 1050 HIV-1 patient samples submitted for HIV drug resistance (HIVDR) testing from January 2022 to June 2023. Evaluation of DRM affecting viral susceptibility to nucleoside/tide reverse transcriptase inhibitors (NRTIs), non-nucleoside reverse transcriptase inhibitors (NNRTIs), protease inhibitors (PIs), and integrase strand transfer inhibitors (INSTIs) was performed using an NGS technology, the Vela Diagnostics Sentosa SQ HIV-1 Genotyping Assay. Results: About 71% of patients were ART naïve and 29% were experienced. Overall, 20% presented with any DRM. The prevalence of TDR and ADR was 12.6% and 41.1%, respectively. M184V, T215S, and M41L mutations for NRTI, K103N for NNRTI, and M46I/L for PIs were frequent in naïve and treated patients. E138K and R263K mutations against INSTIs were more frequent in naïve than treated patients. TDR and ADR to INSTIs were 0.3% and 7%, respectively. Patients aged 50 or over (OR: 1.81, *p* = 0.015), originating from Portuguese-speaking African countries (PALOPs) (OR: 1.55, *p* = 0.050), HIV-1 subtype G (OR: 1.78, *p* = 0.010), and with CD4 < 200 cells/mm^3^ (OR: 1.70, *p* = 0.043) were more likely to present with DRMs, while the males (OR: 0.63, *p* = 0.003) with a viral load between 4.1 to 5.0 Log_10_ (OR: 0.55, *p* = 0.003) or greater than 5.0 Log_10_ (OR: 0.52, *p* < 0.001), had lower chances of presenting with DRMs. Conclusions: We present the first evidence on TDR and ADR to INSTI regimens in followed up patients presenting for healthcare in Portugal. We observed low levels of TDR to INSTIs among ART-naïve and moderate levels in ART-exposed patients. Regimens containing PIs could be an alternative second line in patients with intermediate or high-level drug resistance, especially against second-generation INSTIs (dolutegravir, bictegravir, and cabotegravir).

## 1. Introduction

The advent of highly active antiretroviral therapy (HAART) marked a significant breakthrough in the management of HIV-1 infection, resulting in a notable decrease in both disease-associated morbidity and mortality [1]. However, the rise of drug-resistant virus strains has posed continuous difficulties in the long-term management of the disease [2,3]. The effectiveness of antiretroviral therapy (ART) has been threatened by drug resistance mutations (DRM), ever since the introduction of the first nucleoside reverse transcriptase inhibitor (NRTI) monotherapies and later on with dual nucleoside regimens [4]. Upon identification of a DRM, ART regimens are usually modified accordingly, replacing ineffective drugs with those that are expected to effectively suppress the viral replication [5]. The European multi-center cohort study conducted in the past 10 years and the Portuguese HIV drug resistance (HIVDR) study conducted in 2021 showed that the prevalence of HIV resistance increased over time [6,7,8]. Based on this evidence, the World Health Organization (WHO) stated that further investigation on this subject is necessary in this era of a global scale-up of ART to achieve the 95-95-95 targets [9].

Genotypic testing is widely used to evaluate HIVDR, with Sanger population sequencing being the preferred method. Currently, numerous established methodologies and data analysis instruments are available for this purpose [8]. However, there is a gradual transition occurring in the field from Sanger sequencing to next-generation sequencing (NGS) [10]. Unlike Sanger sequencing, which produces a single consensus sequence for the amplified and sequenced fragment of the HIV-1 genome, NGS techniques generate a large number of sequence reads, often in the range of millions for a single sample [11].

In Portugal, there were 1803 cases of HIV infection diagnosed between 2020 to 2021, with an average rate of 8.7 cases per 100,000 inhabitants affecting mainly men, with the median age at diagnosis being 39.0 years [12]. This report also showed that the majority of new cases were observed in the native population, with a significant but lower proportion of new cases being diagnosed in migrants from Portuguese-speaking African countries (PALOP) and from Brazil (42%) [12]. This study aims to provide a detailed description of the patterns of transmitted drug resistance (TDR) and acquired drug resistance (ADR) in Portugal between 2022 and 2023. Additionally, we identify the most prevalent mutations associated with HIVDR, as well as the factors associated with the emergence of DRMs in HIV-1-infected patients in Portugal.

## 2. Materials and Methods

### 2.1. Study Design and Participants

The resistance data enrolled in this study consisted of patient-level data obtained from the database available at the Western Lisbon Hospital Center (CHLO), National Health System (SNS) in Portugal. The data presented in this study were obtained from isolates that were submitted for testing for HIVDR from 1 January 2022 to 30 June 2023, regardless of treatment history. Genotypic assessment to ascertain the resistance levels towards commercially available nucleoside/tide reverse transcriptase inhibitors (NRTIs), non-nucleoside reverse transcriptase inhibitors (NNRTIs), protease inhibitors (PIs), and integrase strand transfer inhibitors (INSTIs) was performed using the Vela Diagnostics Sentosa SQ HIV-1 Genotyping Assay, an NGS technology.

### 2.2. Next-Generation Sequencing Approach

The Sentosa SQ HIV Genotyping Assay is an NGS-driven comprehensive process, which includes kits designed for RNA extraction, HIV-1 library preparation, and sequencing. It also involves the use of a robotic liquid handling system for RNA extraction and library preparation, Ion Torrent instruments for sequencing, and software for data analysis and reporting. The test can process a maximum of 15 samples simultaneously, with each sample containing 730 μL. The technology performs sequencing of the complete PR gene, the initial 376 amino acids of RT, and the entire IN gene. In the context of this research, we further analyzed the FASTA files generated by the assay that correspond to one consensus nucleotide sequence per isolate. In this sequence, codons that contained mixtures of nucleotides present in variants that corresponded to or exceeded 3.2% of the viral populations were denoted using the International Union of Pure and Applied Chemistry (IUPAC) ambiguity code.

### 2.3. Read Mapping and Variant Calling Analysis

Reads were mapped and aligned against sample-specific reference sequences constructed for the pol-PR/RT/IN genomic region using the Geneious Prime^®^ 2024.0.4. The frequency of each amino acid present in each HIV-1 genomic position was calculated and summarized based on the MINIMAP2 implemented in the HIV Stanford database (http://hivdb.stanford.edu, accessed on 15 December 2023). A list of the amino acids present at these positions and their frequencies was used with the HIVdb program genotypic resistance interpretation algorithm from the Stanford University HIV drug resistance database to infer the levels of susceptibility to PR, RT, and IN. In addition, for each data set, reads spanning amino acid positions (i) 1 to 99 in the protease (HIV-1 strain HXB2 2253 to 2550), (ii) 1 to 268 in the RT (HXB2 2553 to 3353), and (iii) 1 to 273 in the INT (HXB2 4230 to 5046) were extracted for phylogenetic analyses. The consensus sequences were aligned using the Virulign algorithm [13] and manually edited using AliView [14]. The resulting sequence alignments were 1000 and 816 bp long for the PR/RT and INT, respectively. HIV-1 surveillance drug resistance mutations (SDRMs) were inferred using the calibrated population resistance tool in the HIV drug resistance database. Viral subtypes were determined using REGA [15] and comet [16] genotyping tools. The reconstruction of phylogenies using the maximum-likelihood (ML) method was performed in FastTree [17], employing the generalized time-reversible model. The evaluation of statistical support for clades was conducted by employing local support values through the Shimodaira–Hasegawa-like test (SH-test). The Microreact web application was used for the visualization of the phylogenetic tree combined with metadata [18].

### 2.4. Statistical Analysis

The normality of the data distribution was checked using the Shapiro–Wilk test to choose parametric or nonparametric tests. Asymmetric data were presented as median with their interquartile ranges (IQRs) and compared using the Mann–Whitney U test when comparing the two independent groups. Absolute and relative frequencies were presented as descriptive analyses and the association between qualitative variables was evaluated with the Chi-square (X^2^) test. Univariate logistic regression and odds ratio (OR) with their 95% confidence intervals (CIs) were calculated to determine the association between each of the independent (age, gender, sampling origin, HIV-1 subtype, viral load, and CD4 cell count) and each of the dependent (any DRM, NRTI, NNRTI, PI, INST, TDR, and ADR) variables. Variables with *p*-values < 0.2 in the univariate analysis were included in the multivariable model. We used a 5% significance level. Statistical analysis was performed using R version 3.4.1 (R Foundation, Vienna, Austria).

## 3. Results

### 3.1. Demographic and Clinical Characteristics Related to Drug Resistance Mutations

The demographic and laboratory characteristics of the participants are shown in Table 1. A total of 1052 HIV-1 patients were enrolled in this study. The median age of the participants was 41 years old (IQR: 32–50) and 64% were male. Portuguese patients accounted for 46.5%, while migrant patients, such as PALOPs and Brazilians, accounted for 31.3% and 22.2%, respectively. Pure HIV-1 subtypes B (37.8%), G (14.6%), and C (12.3%) were the most prevalent, although other variants (including recombinant forms F1, D, A, and 02_AG) accounted for 35.3%. Around 41% of patients had a viral load Log_10_ greater than 5, and 55% of patients had a CD4 cell count lower than 350 cells/mm^3^, defined as late presenters (LPs). Overall, 70.6% of patients were ART-naïve. The differences in the median ages between ART-experienced (44 years) and ART-naïve (38 years) patients were statistically significant (*p* < 0.001). In addition, statistically significant differences were observed in treatment status between sex (*p* = 0.009) and viral Log_10_ categories (*p* < 0.001).

**Figure 1:**
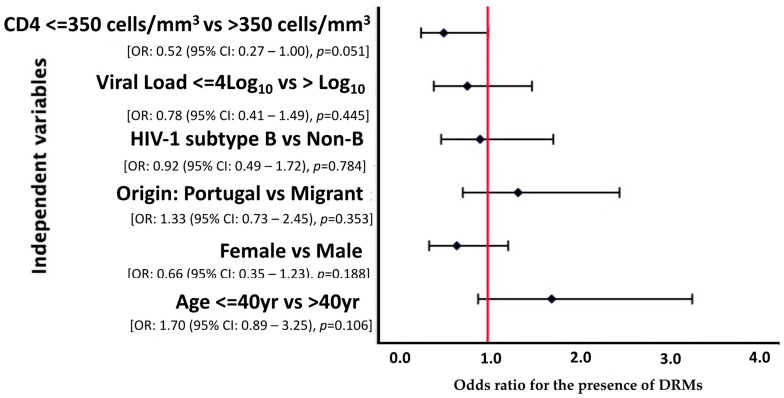
Forest plot of multivariate logistic regression analysis demonstrating OR and 95% CI for the risk factors associated with DRMs among HIV-1 patients presenting for healthcare in Portugal, 2022–2023.

A multivariate analysis was carried out considering variables where *p* < 0.2, however, none of the variables was statistically related to presentation with DRMs (*p* > 0.05). Nonetheless, we observed that patients with a CD4 cell count above 350 cells/mm^3^ were less likely [OR = 0.52 (95% CI: 0.27–1.00), *p* = 0.051] to develop DRMs, with borderline statistical significance (Figure 1).

### 3.2. Characteristics Related to Resistance Mutations

Around 20% (210/1052) of the studied patients had at least one DRM, either for NRTI, NNRTI, PI, or INSTI. Patients aged 50 or over [OR: 1.81 (95% CI: 1.13–2.93), *p* = 0.015] from PALOP [OR: 1.55 (95% CI: 1.0–2.4), *p* = 0. 050], infected with HIV-1 subtype G [OR: 1.78 (95% CI: 1.15–2.74), *p* = 0.010], and with CD4 cell counts up to 200 cells/mm^3^ (late presenters with advanced disease—LPAD) [OR: 1.70 (95% CI: 1.02–2.83), *p* = 0.043] were more likely to present with DRMs compared to the reference group. Male patients [OR: 0.63 (95% CI: 0.46–0.85), *p* = 0.003] had lower chances of presenting with DRMs, and those with a Log_10_ viral load between 4.1 and 5 [OR: 0.55 (95% CI: 0.38–0.82), *p* = 0.003] or greater than 5 [OR: 0.52 (95% CI: 0.35–0.76), *p* < 0.001] also had lower chances of presenting with DRMs when compared to the lower viral load group.

Overall, the prevalence of TDR was 12.6%. The determinants associated with transmitted resistance were male sex [OR: 0.58 (95% CI: 0.36–0.94), *p* = 0.027], viral load between 4.1 and 5.0 [OR: 0.32 (95% CI: 0.17–0.63), *p* < 0.001], and viral load greater than 5.0 [OR: 0.41 (95% CI: 0.22–0.77), *p* = 0.005]. On the other hand, the prevalence of ADR was 41.1%. The determinant associated with acquired resistance was a CD4 cell count up to 200 cells/mm^3^ [OR: 2.48 (95% CI: 1.16–5.30), *p* = 0.019]. Additionally, borderline statistical significance for ADR was observed in patients with CD4 cell counts between 201 and 349 cells/mm^3^ [OR: 2.28 (95% CI: 0.99–5.23), *p* = 0.052], and those age 50 or over [OR: 2.44 (95% CI: 0.97–6.10), *p* = 0.057] (Table 2).

### 3.3. Determinants Related to PR, RT, and INT Drug Resistance Mutations

The DRM description according to the classes of ARVs is shown in Table 3. Overall, the DRMs observed in about 20% (210/1052) of the studied population confer resistance to NRTIs (9.5%), NNRTIs (11.3%), PIs (3.2%), and INSTIs (2.3%). The groups more likely to present with mutations that reduce their susceptibility to NRTIs were patients aged between 30 and 40 years [OR: 2.37 (95% CI: 1.06–5.30), *p* = 0.036], those between 41 and 49 years [OR: 2.74 (95% CI: 1.21–6.22), *p* = 0.016], or aged over 50 years [OR: 3.72 (95% CI: 1.69–8.20), *p* = 0.001], with CD4 cell counts up to 200 cells/mm^3^ [OR: 2.76 (95% CI: 1.41–5.43), *p* = 0.003], and ART experienced [OR: 11.2 (95% CI: 6.63–18.9), *p* < 0.001]. On the other hand, males [OR: 0.60 (95% CI: 0.4–0.92), *p* = 0.018] with a viral load higher than 5.0 Log_10_ [OR: 0.55 (95% CI: 0.32–0.93), *p* = 0.027] were less likely to present with resistance to NRTIs.

Patients from PALOP [OR: 2.03 (95% CI:1.21–3.42), *p* = 0.008] and ART experienced [OR: 3.78 (95% CI: 2.51–5.69), *p* < 0.001] were more likely to harbor resistance against NNRTIs. However, patients with HIV-1B [OR: 0.58 (95% CI: 0.36–0.92), *p* = 0.021], viral load (VL) Log_10_ between 4.1 and 5.0 [OR: 0.58 (95% CI: 0.36–0.94), *p* = 0.026], or VL Log_10_ higher than 5.0 [OR: 0.52 (95% CI: 0.32–0.84), *p* = 0.008] presented with significant lower odds of harboring resistance to NNRTIs. HIV-1G patients [OR: 4.68 (95% CI: 1.7–12.9), *p* = 0.003] had significantly higher odds of drug resistance to PIs than those infected with subtypes B, C, and others.

Concerning INSTIs, those who were ART experienced [OR: 23.2 (95% CI: 5.35–101), *p* < 0.001] had higher odds of presenting with resistance mutation than naïve patients. Those with viral loads ranging from 4.1 to 5.0 Log_10_ [OR: 0.27 (95% CI: 0.1–0.71), *p* = 0.008] or VL Log_10_ higher than 5.0 [OR: 0.08 (95% CI: 0.02–0.35), *p* < 0.001] were less likely to harbor resistance to integrase inhibitors.

**Figure 2:**
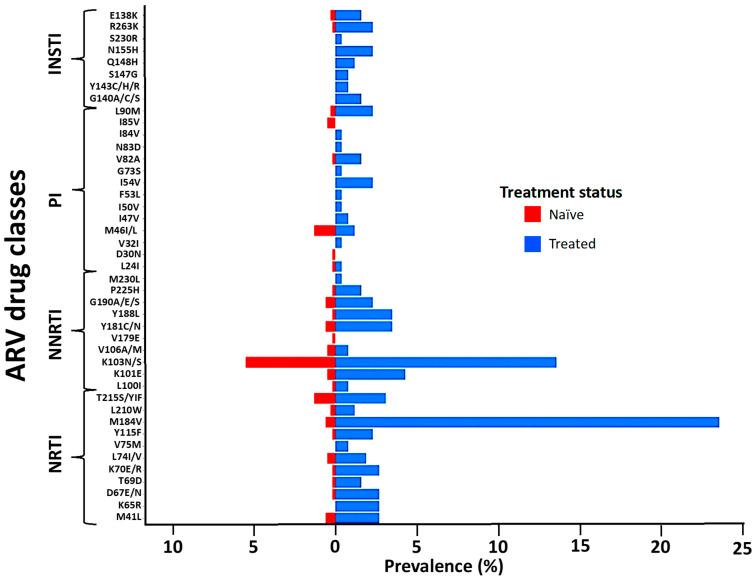
Distribution of the observed DRMs according to the ARV drug class and treatment status. Abbreviations: NRTIs, nucleoside reverse transcriptase inhibitors; NNRTIs, non-nucleoside reverse transcriptase inhibitors; PIs, protease inhibitors; INSTIs, integrase strand inhibitors. Evaluation of drug resistance mutations affecting susceptibility to NRTIs, NNRTIs, PIs, and INSTIs, based on the WHO SDRM list updated in 2009.

**Figure 3:**
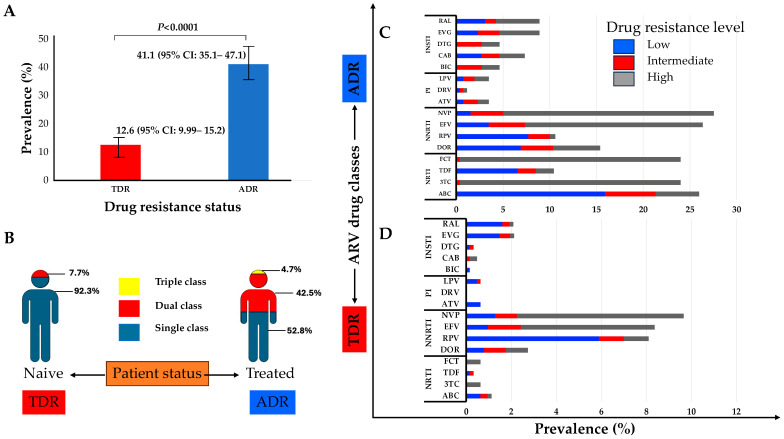
(**A**) Prevalence of transmitted drug resistance (TDR) and of acquired drug resistance (ADR); (**B**) proportion of TDR and ADR for single to multiple class resistance to ARV; (**C**) clinical impact of the DRMs on ART-experienced patients; (**D**) clinical impact of the DRMs on ART-naïve patients. Scores of low-level (scores 2 and 3, blue), intermediate-level (score 4, red), or high-level (score 5, grey) resistance were used to predict phenotypic resistance. Phenotypic characterization was carried out on the HIV drug resistance database website at Stanford University (https://hivdb.stanford.edu/, accessed on 15 December 2023). Abbreviations: ADR, acquired drug resistance; TDR, transmitted drug resistance; NRTIs, nucleoside/tide reverse transcriptase inhibitors; NNRTIs, non-nucleoside reverse transcriptase inhibitors; PIs, protease inhibitors; INSTIs, integrase strand inhibitors; RAL, raltegravir; EVG, elvitegravir; DTG, dolutegravir; CAB, cabotegravir; BIC, bictegravir; LVP, lopinavir; DRV, darunavir; ATV, atazanavir; NVP, nevirapine; EFV, efavirenz; DOR, doravirine; FTC, emtricitabine; TDF, tenofovir; 3TC, lamivudine; ABC, abacavir.

The M184V, T215S, and M41L mutations of the NRTI class were the most prevalent in naïve and treated patients. Similarly, the K103N mutation of the NNRTI class and M46I/L of the PIs were the most frequent in both naïve and treated groups. The E138K and R263K mutations of the INSTI class were the only ones observed in naïve patients and were also the most prevalent in treated patients, followed by the N155H and G140A mutations (Figure 2). The prevalence of TDR was 12.6% (95% CI: 9.99–15.2) while ADR was 41.1% (95% CI: 35.1–47.1) (Figure 3A). Some of the TDR patients presented with resistance to single (92.3%) and dual (7.7%) ARV classes, while ADR patients presented with resistance to single (53.8%), double (42.5%), and triple (4.7%) classes (Figure 3B). The distribution of resistance to NRTIs among ART-experienced patients according to the type of ARV drug ranged from around 10% to 26%, with tenofovir-TDF (10%) showing the lowest resistance, compared to emtricitabine-FTC (24%), lamivudine-3TC (24%), and abacavir-ABC (26%). NNRTI resistance was between 15% and 28% in ART-experienced patients, with efavirenz-EFV (26%) and nevirapine-NVP (28%) experienced, and doravirine-DOR (15%). Resistance to PIs was below 5% in patients who were ARV experienced, with darunavir-DRV (1.4%) showing the lowest prevalence of resistance compared to atazanavir-ATV (3.5%) and lopinavir-LPV (3.5%). In the INSTI-experienced patients, the prevalence ranged between 5% and 9%, with raltegravir-RAL (8.9%), elvitegravir-EVG (8.9%), and cabotegravir-CAB (7.3%) having the highest prevalence. It is worth mentioning that although the prevalence of DRMs in patients experienced with dolutegravir-DTG (4.6%) and bictegravir-BIC (4.6%) is lower compared to other ARVs in the same class, all such patients presented with intermediate and high-level resistance (Figure 3C).

In ART-naïve patients, resistance to NRTIs was less than 2%, with high-level resistance to FTC (0.64%) and 3TC (0.64%), while 1% of patients showed DR to ABC (1.1%), though this was mostly low and intermediate resistance (0.32%). No high-level resistance to TDF was found in naïve patients. Drug resistance to NNRTIs was present in between 3% and 10% of naïve patients, with NVP (9.7%) and EFV (8.4%) being the drugs with the highest prevalence of resistance compared to DOR (2.7%). Resistance to PIs in naïve patients was less than 1%, and, of these, high levels of resistance were not observed. No resistance was found to DRV, while for LPV and ATV, it was present at a prevalence of 0.6% each. The prevalence of drug resistance to INSTIs in naïve patients varied between 0.2% to 2%, with resistance to RAL (2%) and EVG (2%), although with a low prevalence of high-level resistance (0.2%) to each. Notably, the prevalence of high-level (0.32%) and intermediate-level (0.16%) resistance is noteworthy for CAB, although at a low prevalence of 0.5%. The prevalence of DTG in naïve patients was 0.32%, with low-level (0.2%) and intermediate-level (0.2%) resistance. The prevalence of drug resistance to BIC in naïve patients was 0.2%, all with low-level resistance (Figure 3D).

### 3.4. Country of Origin and DRM Distribution by Phylogenetic Tree

**Figure 4:**
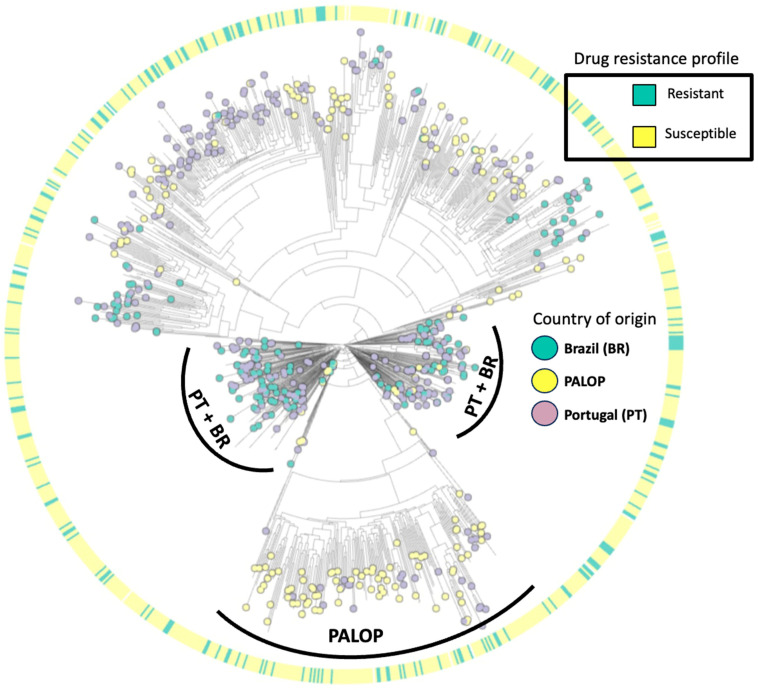
Maximum-likelihood phylogenetic tree of HIV-1 isolates from patients presenting for healthcare in Portugal from 2022–2023.

The maximum-likelihood phylogenetic tree (Figure 4) illustrates the distribution of HIV-1 subtypes detected in patients presenting for healthcare in Portugal. Our phylogenetic analysis indicates that the predominant HIV-1 is subtype B (37.8%, 398/1052), followed by subtypes G (14.6%, 154/1052), C (12.3%, 129/1052), CRF02_AG (10.2%, 107/1052), A (6.7%, 70/1052), F1 (6.6%, 69/1052), others (0.7%, 7/1052), and recombinants (10.6%, 112/1052). Overall, 32.9% of patients harboring at least one DRM had HIV-1 subtype B, 21.4% had subtype G, 12.4% had subtype C, and 33% had other subtypes. Regarding the country of origin, we detected viral clusters of sequences from PALOPs, while sequences from autochthonous (Portuguese population) patients had more similarity with sequences from patients from Brazil.

## 4. Discussion

The dissemination of DRMs remains a significant public health concern in the current era of ART, particularly in relation to emerging infection-preventing approaches such as pre-exposure prophylaxis (PrEP) [4]. According to recent records, the HIV epidemic in Portugal has changed significantly in recent years. In the latest national epidemiological report from 2021 to 2022, a total of 804 new HIV infections were reported, mostly in men, with the highest diagnosis rate observed in the 25–29 age group. The majority of new infections (51.7%) were diagnosed in the Portuguese population, with sexual transmission route being the most common, from which, men who had sex with men (MSM) accounted for 61.8% of the infections [12].

Our study represents an updated assessment of TDR and ADR to ARV in Portugal using an NGS approach. A total of 1052 patients were enrolled, 70% of whom were newly diagnosed and ART naïve, while the remaining 30% had previous received ART. Our study has shed light on the prevalence of resistance to INSTIs, which is crucial to inform guidelines for first-line ART regimens in Portugal. This study described the prevalence of TDR/ADR and the HIV-1 subtype in individuals presenting for healthcare in Portugal in the period 2022–2023 and further analyzed the phylogenetic distribution of HIV according to the country of origin and the presence of DRMs. The results showed that the rate of TDR was 12.5%, considered moderate according to the WHO [19], and slightly higher than observed in the last study on TDR in Portugal [20]. A study involving 26,973 HIV-1-infected patients from the EuResist Integrated Database (EIDB) between 1981 and 2019 covering Italy, Germany, Spain, Sweden, Belgium, Portugal, and Luxembourg identified a similar overall TDR rate (12.8%) concerning PIs, NRTIs, and NNRTIs, indicating that it has remained stable in recent years. The possible hypothesis is that the NGS approach detects minority populations (<20%) [10] that cannot be detected by the Sanger used in the last study. Around 55% of the new HIV-1 diagnoses were related to late presentation (LP), which would explain the identification of mutations such as K103N in patients treated with previous regimens containing Efavirenz. The long-lasting presence of NNRTI mutations, even after stopping the use of NNRTIs, is frequently seen and may be attributed to the little overall effect on the viral fitness of mutations like K103N [21]. On the other hand, our results were not expected, since Portugal has adopted first-line ART regimen recommendations containing high genetic barrier drugs, such as DTG and more recently BIC, which should significantly decrease the TDR prevalence.

The detection of SDRMs in the present study was consistent with other recent studies published in Europe [6,7,8], making our findings one of the more up-to-date studies including the surveillance of resistance to INSTIs. The rate of SDRMs conferring resistance to NRTIs remained stable and similar to a previous study conducted in Portugal [7]. Interestingly, M184V continues to be transmitted at a very low proportion, with most SDRMs being transmitted as singletons. On the other hand, SDRMs conferring resistance to NNRTIs were 1.5 times higher in our study (7.7%) compared to the last study conducted in Portugal (4.9%), with K103N being the most prevalent. The reason for this increase in prevalence is not clear and is intriguing, given that NNRTIs have not been used as first-line in Portugal since 2017. However, the incoming migrants from PALOPs, where NNRTIs were used until more recently, could help explain this finding.

On the other hand, the prevalence of drug resistance to PIs was lower than observed in the previous study (2.5% vs. 3.9%) [7]. As expected, drug resistance to INSTIs in naïve patients was very low (0.3%), and these findings corroborate data from the European literature, where the prevalence of TDR to INSTIs ranges from 0.2% to 1.7% [22,23,24,25], with E138K and R263K being more frequently identified, which can be selected by the first-line ART regimen currently adopted in Portugal.

The prevalence of ADR was approximately 40%, which is in line with other studies in Portugal and Europe. A previous study in Portugal covering ADR to three drug classes (NNRTI, NRTI, and PI) showed a decreasing trend in ADR over the last two decades [20]. This trend has been consistently observed in other European countries, such as Switzerland, Italy, Germany, Spain, Sweden, Belgium, and Luxembourg [8]. Possible explanations for the decrease in ADR across Europe could include some or all of these factors: (i) a higher genetic barrier in currently used regimens containing INSTIs, (ii) fewer tablets and/or simplified ART regimens, (iii) reduced drug toxicity, and (iv) improved patient adherence.

Our subtyping analysis showed high genetic diversity, with a higher prevalence of subtypes B and G. These findings are in line with the previous literature which points to a complex distribution of HIV-1 subtypes in Portugal with a high prevalence of subtype B, followed by G [26,27,28]. Our phylogenetic tree aimed to indicate patterns of clustering of viral drug resistance strains and country of origin. The sequences harboring DRMs had a homogeneous distribution across the tree, indicating a lack of association with subtype or country of origin. On the other hand, the distribution of sequences according to country of origin was heterogeneous, as sequences from PALOP immigrants were more likely to cluster together while sequences from Brazilian migrants showed remarkable clustering patterns with autochthonous patients. These data are in line with the previous study published by our research team on the patterns of acquisition of HIV-1 infection among the migrant population in Portugal, where PALOP migrants shared transmission clusters indicating intra-community transmission of non-B subtypes. On the other hand, immigrants from Brazil were more likely to belong to transmission clusters of Portuguese origin [29]. We understand that the phylogenetic model carried out in the present study did not aim to determine transmission clusters. However, it allowed us to provide insights into genetic variability, as well as the clustering pattern of DRMs and the country of origin.

The present study had some limitations. First, the studied population was restricted to the Lisbon metropolitan area, which corresponds to about 52% of new infections in Portugal and therefore does not represent the epidemic at a national level. Second, there was missing data on the clinical and sociodemographic characteristics of the study participants, which reduced the statistical power of the study. Third, the sequenced fragment does not cover the complete RT gene, which prevented surveillance of the N348I mutation. This mutation is not listed by the WHO as an SDRM, having little clinical relevance and low potential for resistance to nevirapine, which is not part of the preferred therapeutic regimen in Portugal. Finally, there was an imbalance in the database between naïve and treated patients, which could lead to a bias in the rates of TDR and ADR. Nonetheless, our findings provided an update on the HIV molecular epidemiology contributing to the understanding of circulating HIV-1 DRMs among autochthonous and/or migrants affected by the HIV epidemic in Portugal.

## 5. Conclusions

We observed 0.3% of major resistance mutations to INSTIs in ART-naïve patients and 7% in ART-exposed patients. Interestingly, it was observed that regimens containing PIs could serve as alternative care for patients with intermediate or high-level drug resistance, especially against second-generation INSTIs. The resistance patterns did not differ between autochthonous (Portuguese population) and migrant patients. However, phylogenetic clustering between patients from Portugal and Brazil suggests common and continuous transmission clusters, while monophyletic clustering between immigrants from PALOP indicates disaggregated and independent HIV-1 transmission patterns. Since INSTIs have now been scaled up globally, continuous surveillance of INSTI resistance is crucial.

## Figures and Tables

**Table 1:** Demographic and clinical characteristics related to antiretroviral treatment status among HIV-1 patients presenting for healthcare in Portugal, 2022–2023.

Independent Variable	N (%)	Treatment Status		
		Experienced (%)	Naïve (%)	*p*-Value
**Overall (Missing = 173)**	1052 (100)	258 (29.4)	621 (70.6)	
**Demographic**				
Age–yr–Median (IQR)	41 (32–50)	44.0 (35.0–52.0)	38.0 (30.5–48.0)	<0.001 *
Age distribution (Missing = 28)				
<30 yr	200 (19.5)	30 (11.8)	138 (22.4)	<0.001 *
30–40 yr	312 (30.5)	68 (26.7)	203 (32.9)	
41–49 yr	244 (23.8)	74 (29.0	133 (21.6)	
≥50 yr	268 (26.2)	83 (32.5)	143 (23.2)	
Sex (Missing = 26)				
Female	369 (36.0)	107 (41.8)	201 (32.5)	0.009 *
Male	657 (64.0)	149 (58.2)	417 (67.5)	
Sampling origin (Missing = 445)				
Portugal	282 (46.5)	85 (47.0)	186 (46.0)	0.548
Brazil	135 (22.2)	36 (19.9)	96 (23.8)	
PALOP	190 (31.3)	60 (33.1)	122 (30.2)	
**Clinical**				
HIV-1 Subtype				
B	398 (37.8)	99 (38.4)	239 (38.5)	0.223
G	154 (14.6)	47 (18.2)	81 (13.0)	
C	129 (12.3)	28 (10.9)	78 (12.6)	
Others	371 (35.3)	84 (32.6)	223 (35.9)	
Viral load (Missing = 53)	4.81 (4.08–5.45)	4.23 (3.17–5.10)	4.96 (4.38–5.73)	<0.001 *
<4.0 Log_10_	235 (23.5)	113 (44.5)	82 (13.9)	<0.001 *
4.1 to 5.0 Log_10_	359 (35.9)	76 (29.9)	234 (39.7)	
>5.0 Log_10_	405 (40.5)	65 (25.6)	274 (46.4)	
CD4–mm^3^, Median (IQR)	312 (148–534)	273 (137–469)	339 (156–550)	0.133
≤200 mm^3^	136 (33.4)	53 (34.9)	83 (32.5)	0.220
201–349 mm^3^	87 (21.4)	38 (25.0)	49 (19.2)	
≥350 mm^3^	184 (45.2)	61 (40.1)	123 (48.2)	

* The results were statistically significant for the Mann–Whitney U Test, Chi-square test, or univariate analysis (*p* < 0.05).

**Table 2:** Demographic and clinical characteristics related to acquired and transmitted drug resistance among HIV-1 patients presenting for healthcare in Portugal, 2022–2023.

Independent Variable	Any DRM (N = 1052)	OR (95% CI)	*p*-Value	TDR (Naive = 621)	OR (95% CI)	*p*-Value	ADR (Treated = 258)	OR (95% CI)	*p*-Value
Overall	210 (20.0)			78 (12.6)			106 (41.1)		
Age distribution, n (%)									
<30 yo	30 (14.7)	1.00		17 (21.8)	1.00		8 (7.6)	1.00	
30–40 yo	59 (28.9)	1.32 (0.82–2.14)	0.256	28 (35.9)	1.14 (0.60–2.17)	0.693	29 (27.6)	2.05 (0.80–5.24)	0.136
41–49 yo	50 (24.5)	1.46 (0.89–2.40)	0.135	17 (21.8)	1.04 (0.51–2.14)	0.908	29 (27.6)	1.77 (0.70–4.51)	0.230
≥50 yo	65 (31.9)	1.81 (1.13–2.93)	0.015 *	16 (20.5)	0.90 (0.43–1.86)	0.769	39 (37.1)	2.44 (0.97–6.10)	0.057
Gender, n (%)									
Female	92 (44.9)	1.00		34 (43.6)	1.00		48 (45.3)	1.00	
Male	113 (55.1)	0.63 (0.46–0.85)	0.003 *	44 (56.4)	0.58 (0.36–0.94)	0.027 *	58 (54.7)	0.78 (0.47–1.30)	0.342
Sampling origin, n (%)									
Portugal	54 (41.5)	1.00		21 (42.9)	1.00		31 (40.8)	1.00	
Brazil	25 (19.2)	0.96 (0.57–1.62)	0.878	10 (20.4)	0.91 (0.41–2.03)	0.824	14 (18.4)	1.11 (0.50–2.47)	0.801
PALOP	51 (39.2)	1.55 (1.00–2.40)	0.050	18 (36.7)	1.36 (0.69–2.67)	0.373	31 (40.8)	1.86 (0.95–3.65)	0.070
HIV-1 Subtype, n (%)									
B	69 (32.9)	0.90 (0.63–1.30)	0.581	26 (33.3)	0.82 (0.47–1.43)	0.481	36 (34.0)	0.84 (0.46–1.53)	0.569
G	45 (21.4)	1.78 (1.15–2.74)	0.010 *	16 (20.5)	1.65 (0.84–3.22)	0.146	21 (19.8)	1.19 (0.58–2.44)	0.640
C	26 (12.4)	1.09 (0.66–1.79)	0.749	7 (9.0)	0.66 (0.28–1.57)	0.348	15 (14.2)	1.70 (0.71–4.01)	0.229
Others	70 (33.3)	1.00		29 (37.2)	1.00		34 (32.1)	1.00	
Viral load, n (%)									
≤4.0	67 (33.3)	1.00		20 (27.0)	1.00		41 (39.4)	1.00	
4.1 to 5.0	65 (32.3)	0.55 (0.38–0.82)	0.003 *	22 (29.7)	0.32 (0.17–0.63)	<0.001 *	38 (36.5)	1.76 (0.97–3.17)	0.062
>5.0	69 (34.3)	0.52 (0.35–0.76)	<0.001 *	32 (43.2)	0.41 (0.22–0.77)	0.005 *	25 (24.0)	1.10 (0.59–2.06)	0.772
CD4, n (%)									
≤200 mm^3^	41 (40.6)	1.70 (1.02–2.83)	0.043 *	12 (37.5)	1.05 (0.48–2.34)	0.897	29 (42.0)	2.48 (1.16–5.30)	0.019 *
201–349 mm^3^	23 (22.8)	1.44 (0.79–2.60)	0.236	3 (9.4)	0.41 (0.11–1.46)	0.167	20 (29.0)	2.28 (0.99–5.23)	0.052
≥350 mm^3^	37 (36.6)	1.00		17 (53.1)	1.00		20 (29.0)	1.00	

* The results were statistically significant for univariate analysis (*p* < 0.05).

**Table 3:** Determinants related to PR, RT, and INST drug resistance mutations among HIV-1 patients presenting for healthcare in Portugal, 2022–2023.

Independent Variable	N (%)	NRTI			NNRTI			PI			INSTI		
		N (%)	OR (95% CI)	*p*-Value	N (%)	OR (95% CI)	*p*-Value	N (%)	OR (95%CI)	*p*-Value	N (%)	OR (95% CI)	*p*-Value
**Overall**	1052 (100)	100 (9.5)			119 (11.3)			34 (3.2)			24 (2.3)		
Age distribution													
<30 yr	200 (19.5)	8 (8.2)	1.00			1.00		7 (20.6)	1.00		0 (0.0)	0.0 (0.0–0.0)	0.995
30–40 yr	312 (30.5)	28 (28.9)	2.37 (1.06–5.30)	0.036 *	19 (16.1)	1.13 (0.62–2.04)	0.694	8 (23.5)	0.73 (0.26–2.03)	0.542	5 (25.0)	0.86 (0.25–2.99)	0.808
41–49 yr	244 (23.8)	25 (25.8)	2.74 (1.21–6.22)	0.016 *	33 (28.0)	1.44 (0.78–2.62)	0.236	5 (14.7)	0.58 (0.18–1.85)	0.354	10 (50.0)	2.25 (0.76–6.67)	0.144
≥50 yr	268 (26.2)	36 (37.1)	3.72 (1.69–8.20)	0.001 *	32 (27.1)	1.38 (0.76–2.51)	0.283	14 (41.2)	1.52 (0.6–3.84)	0.376	5 (25.0)	1.00	
Sex													
Female	369 (36.0)	46 (46.9)	1.00		51 (42.9)	1.00		16 (47.1)	1.00		8 (40.0)	1.00	
Male	657 (64.0)	52 (53.1)	0.60 (0.40–0.92)	0.018 *	68 (57.1)	0.72 (0.49–1.06)	0.097	18 (52.9)	0.62 (0.31–1.23)	0.174	12 (60.0)	0.84 (0.34–2.07)	0.704
Sampling origin													
Portugal	282 (46.5)	29 (42.6)	1.00		30 (38.0)	1.00		7 (36.8)	1.00		5 (41.7)	1.00	
Brazil	135 (22.2)	15 (22.1)	1.09 (0.56–2.11)	0.797	12 (15.2)	0.82 (0.41–1.66)	0.579	6 (31.6)	1.83 (0.60–5.55)	0.287	2 (16.7)	0.83 (0.16–4.35)	0.829
PALOP	190 (31.3)	24 (35.3)	1.26 (0.71–2.24)	0.429	37 (46.8)	2.03 (1.21–3.42)	0.008 *	6 (31.6)	1.28 (0.42–3.87)	0.661	5 (41.7)	1.50 (0.43–5.24)	0.528
HIV-1 Subtype													
B	398 (37.8)	39 (39.0)	1.37 (0.77–2.44)	0.278	32 (26.9)	0.58 (0.36–0.92)	0.021 *	16 (47.1)	2.55 (0.99–6.58)	0.053	8 (33.3)	0.83 (0.32–2.16)	0.696
G	154 (14.6)	20 (20.0)	1.03 (0.53–2.0)	0.927	21 (17.6)	1.04 (0.6–1.80)	0.895	11 (32.4)	4.68 (1.70–12.9)	0.003 *	2 (8.3)	0.53 (0.11–2.48)	0.419
C	129 (12.3)	13 (13.0)	0.75 (0.45–1.25)	0.270	17 (14.3)	1.0 (0.55–1.8)	0.993	1 (2.9)	0.48 (0.57–3.99)	0.493	5 (20.8)	1.62 (0.53–4.93)	0.394)
Others	371 (35.3)	28 (28.0)	1.00		49 (41.2)	1.00		6 (17.6)	1.00		9 (37.5)	1.00	
Viral load													
<4.0	235 (23.5)	30 (30.9)	1.00		39 (34.2)	1.00		11 (33.3)	1.00		14 (63.6)	1.00	
4.1 to 5.0	359 (35.9)	37 (38.1)	0.79 (0.47–1.31)	0.355	37 (32.5)	0.58 (0.36–0.94)	0.026 *	11 (33.3)	0.64 (0.27–1.51)	0.311	6 (27.3)	0.27 (0.10–0.71)	0.008 *
>5.0	405 (40.5)	30 (30.9)	0.55 (0.32–0.93)	0.027 *	38 (33.3)	0.52 (0.32–0.84)	0.008 *	11 (33.3)	0.57 (0.24–1.33)	0.194	2 (9.1)	0.08 (0.02–0.35)	<0.001 *
CD4 cell count													
≤200 mm^3^	136 (33.4)	27 (48.2)	2.76 (1.41–5.43)	0.003 *	23 (39.7)	1.57 (0.83–2.97)	0.167	4 (26.7)	0.76 (0.22–2.66)	0.669	5 (41.7)	1.36 (0.39–4.79)	0.634
201–349 mm^3^	87 (21.4)	14 (25.0)	2.17 (0.99–4.72)	0.051	14 (24.1)	1.5 (0.72–3.10)	0.280	4 (26.7)	1.22 (0.35–4.30)	0.752	2 (16.7)	0.85 (0.16–4.45)	0.844
≥350 mm^3^	184 (45.2)	15 (26.8)	1.00		21 (36.2)	1.00		7 (46.7)	1.00		5 (41.7)	1.00	
Treatment status													
Experienced	258 (29.4)	70 (77.8)	11.2 (6.63–18.9)	<0.001 *	62 (56.2)	3.78 (2.51–5.69)	<0.001 *	12 (42.9)	1.85 (0.86–3.96)	0.116	18 (90.0)	23.2 (5.35–101)	<0.001 *
Naïve	621 (70.6)	20 (22.2)	1.00		48 (43.6)	1.00		16 (57.1)	1.00		2 (10.0)	1.00	

Abbreviations: NRTIs, nucleoside reverse transcriptase inhibitors; NNRTIs, non-nucleoside reverse transcriptase inhibitors; PIs, protease inhibitors; INSTIs, integrase strand transfer inhibitors; OR, odds ratio; CI, confidence interval. * The results were statistically significant for univariate analysis (*p* < 0.05).

## Data Availability

Data are unavailable due to privacy or ethical restrictions.
